# Activation of Sterol Regulatory Element Binding Factors by Fenofibrate and Gemfibrozil Stimulates Myelination in Zebrafish

**DOI:** 10.3389/fphar.2016.00206

**Published:** 2016-07-11

**Authors:** Yoshifumi Ashikawa, Yuhei Nishimura, Shiko Okabe, Shota Sasagawa, Soichiro Murakami, Mizuki Yuge, Koki Kawaguchi, Reiko Kawase, Toshio Tanaka

**Affiliations:** ^1^Department of Molecular and Cellular Pharmacology, Pharmacogenomics and Pharmacoinformatics, Mie University Graduate School of MedicineTsu, Japan; ^2^Department of Systems Pharmacology, Mie University Graduate School of MedicineTsu, Japan; ^3^Mie University Medical Zebrafish Research CenterTsu, Japan; ^4^Department of Omics Medicine, Mie University Industrial Technology Innovation InstituteTsu, Japan; ^5^Department of Bioinformatics, Mie University Life Science Research CenterTsu, Japan

**Keywords:** SREBFs, fenofibrate, gemfibrozil, oligodendrocytes, myelination, comparative transcriptomics, zebrafish, systems pharmacology

## Abstract

Oligodendrocytes are major myelin-producing cells and play essential roles in the function of a healthy nervous system. However, they are also one of the most vulnerable neural cell types in the central nervous system (CNS), and myelin abnormalities in the CNS are found in a wide variety of neurological disorders, including multiple sclerosis, adrenoleukodystrophy, and schizophrenia. There is an urgent need to identify small molecular weight compounds that can stimulate myelination. In this study, we performed comparative transcriptome analysis to identify pharmacodynamic effects common to miconazole and clobetasol, which have been shown to stimulate myelination by mouse oligodendrocyte progenitor cells (OPCs). Of the genes differentially expressed in both miconazole- and clobetasol-treated mouse OPCs compared with untreated cells, we identified differentially expressed genes (DEGs) common to both drug treatments. Gene ontology analysis revealed that these DEGs are significantly associated with the sterol biosynthetic pathway, and further bioinformatics analysis suggested that sterol regulatory element binding factors (SREBFs) might be key upstream regulators of the DEGs. *In silico* screening of a public database for chemicals associated with SREBF activation identified fenofibrate, a peroxisome proliferator-activated receptor α (PPARα) agonist, as a drug that increases the expression of known SREBF targets, raising the possibility that fenofibrate may also stimulate myelination. To test this, we performed *in vivo* imaging of zebrafish expressing a fluorescent reporter protein under the control of the myelin basic protein (mbp) promoter. Treatment of zebrafish with fenofibrate significantly increased expression of the fluorescent reporter compared with untreated zebrafish. This increase was attenuated by co-treatment with fatostatin, a specific inhibitor of SREBFs, confirming that the fenofibrate effect was mediated via SREBFs. Furthermore, incubation of zebrafish with another PPARα agonist, gemfibrozil, also increased expression of the mbp promoter-driven fluorescent reporter in an SREBF-dependent manner. These results suggest that activation of SREBFs by small molecular weight compounds may be a feasible therapeutic approach to stimulate myelination.

## Introduction

Oligodendrocytes are major myelinating cells of the central nervous system (CNS) and are thus critical to proper neuronal functioning. However, they are also an extremely vulnerable cell type, and CNS myelin abnormalities are found in a variety of neurological disorders (reviewed in Chew and DeBoy, 2015), including white matter pathologies associated with brain injury, endocrine and metabolic abnormalities, and psychiatric and neurodegenerative conditions. The aberrant myelination associated with these heterogeneous pathologies can result from the production of myelin with abnormal structure, the virtual absence of myelin, and damage to existing myelin by insults such as oxidative stress, mechanical injury, and inflammation. Ultimately, the loss of normal myelination leads to defective impulse conduction along the nerve fiber.

Current therapeutic approaches are aimed at reducing demyelination by immune-, excitotoxic-, and oxidative stress-mediated injury and at promoting myelination through cell engraftment, direct protection of endogenous oligodendrocytes, and enhancement of OPC activity. However, there are some reports of effective non-immune-based remyelinating therapeutics, including benztropine (Deshmukh et al., 2013), miconazole (Najm et al., 2015), clobetasol (Najm et al., 2015; Porcu et al., 2015), and halcinonide (Porcu et al., 2015). Benztropine stimulates myelination by antagonizing the M1/M3 muscarinic receptor (Deshmukh et al., 2013), whereas miconazole and clobetasol stimulate myelination, at least in part, through mitogen-activated protein kinase and glucocorticoid receptor signaling, respectively (Najm et al., 2015). Clobetasol and halcinonide have also been shown to stimulate myelination through activation of Smoothened in the hedgehog signaling pathway and retinoid X receptor γ (RXRγ) (Porcu et al., 2015). This common mechanism of action suggests the existence of convergent pathways through which the drugs may stimulate myelination.

In this study, we performed comparative transcriptome analysis of mouse epiblast stem cell-derived OPCs (mEpiSC-OPCs) treated with miconazole and clobetasol to identify common mechanisms underlying myelination. We were able to identify a number of genes regulated in common by miconazole and clobetasol. Bioinformatic analysis of these genes revealed that SREBFs may be involved in myelination induced by miconazole and clobetasol, raising the possibility that other SREBF-activating drugs may also stimulate myelination. *In silico* screening of a public database revealed that the peroxisome proliferator-activated receptor α (PPARα) agonist fenofibrate can activate SREBFs, which prompted us to examine the drug’s effect on myelination *in vivo*. Using *in vivo* imaging of zebrafish expressing a fluorescent reporter protein driven by the mbp promoter, we confirmed that fenofibrate and a second PPARα agonist, gemfibrozil, could increase mbp promoter activity in a SREBF-dependent manner. Quantitative PCR (qPCR) analysis demonstrated that the PPARα agonists increased the expression of two genes downstream of SREBFs and mbp. These results suggest that activation of SREBFs may be a convergent pathway for drugs that stimulate myelination.

## Materials and Methods

### Ethics Statement

This study was carried out in strict accordance with Japanese law [The Humane Treatment and Management of Animals (2014), Standards Relating to the Care and Management of Laboratory Animals and Relief of Pain (2013), and the Guidelines for Proper Conduct of Animal Experiments (2006) (Science Council of Japan, 2006; Ministry of the Environment of Japan, 2013, 2014)]. All efforts were made to minimize animal suffering. Mie University Institutional Animal Care and Use Committee guidelines state that no approval is required for experiments using zebrafish.

### Compounds

Fenofibrate and gemfibrozil were obtained from Tokyo Chemical Industry (Tokyo, Japan). Methimazole, propylthiouracil, thyroxine, and fatostatin were obtained from Sigma (St. Louis, MO, USA). Stock solutions of these chemicals were prepared by dissolving in dimethyl sulfoxide (Nacalai Tesque, Kyoto, Japan). 2-Phenoxyethanol was obtained from Wako Chemical (Osaka, Japan).

### Comparative Transcriptome Analysis of mEpiSC-OPCs Treated with Miconazole or Clobetasol

To identify genes related to the mechanisms of miconazole- and clobetasol-induced myelination, we downloaded a transcriptome dataset (GSE63804) from Gene Expression Omnibus (Barrett et al., 2009), which was derived from an analysis of the effects of miconazole and clobetasol on the enhancement of myelination by mEpiSC-OPCs (Najm et al., 2015). The raw data from GSE63804 were processed according to previous reports (Trapnell et al., 2009; Liao et al., 2013, 2014). A count-based differential expression analysis was performed using “TCC” (Sun et al., 2013) to identify DEGs in mEpiSC-OPCs treated with miconazole or clobetasol for 2, 6, or 12 h compared with untreated mEpiSC-OPCs, using a false discovery rate of 20% as the threshold. The murine gene symbols of the DEGs were converted to those of the human orthologs using Life Science Knowledge Bank (World Fusion, Tokyo, Japan). The lists of DEGs are shown in Supplementary Tables S1(1–6). DEGs common to mEpiSC-OPCs treated with miconazole and clobetasol are shown in Supplementary Table S2.

### Bioinformatic Analysis of Genes Regulated by Both Miconazole and Clobetasol

To identify the biological pathways enriched in the DEGs regulated by both miconazole and clobetasol in mEpiSC-OPCs, we used ClueGO (Bindea et al., 2009) in Cytoscape (Shannon et al., 2003) with the default settings. The biological pathways significantly enriched in the DEGs are shown in Supplementary Tables S3-1 (6 h) and S3-2 (12 h).

To identify TFs potentially regulating the common DEGs, we used iRegulon (Janky et al., 2014) in Cytoscape (Shannon et al., 2003). iRegulon exploits the fact that genes co-regulated by the same TF contain common TF binding sites, and uses gene sets derived from ENCODE ChIP-seq data (Gerstein et al., 2012; Janky et al., 2014). The predicted TFs with normalized enrichment scores >5 are shown in Supplementary Tables S4-1 (6 h) and S4-2 (12 h).

### Identification of Chemicals That Increase Expression of Genes Targeted by SREBFs

To identify chemicals that have been reported to increase the expression of genes regulated by SREBFs, we searched the Comparative Toxicogenomics Database (Davis et al., 2015). The database has been successfully used for *in silico* prediction of biological pathways associated with metal exposure and developmental disorders, followed by experimental validation of the pathway prediction (Ahir et al., 2013). We searched for compounds that increased the expression of *HMGCR* and *SCD.* A list of chemicals identified by the *in silico* screening are shown in Supplementary Tables S5(1) (*SCD*) and Supplementary Table S5(2) (*HMGCR*). A list of FDA-approved drugs common to Supplementary Tables S5(1,2) is shown in Supplementary Table S5(3).

### Zebrafish Strains

We used an albino zebrafish line (Kelsh et al., 1996) obtained from the Max Planck Institute for Developmental Biology (Tübingen, Germany) to make transgenic Tg (mbp: mCitrine) zebrafish, which express the cyan fluorescent protein mCitrine under the control of the mbp promoter (Jung et al., 2010), allowing us to visualize myelin-producing cells. The promoter and part of the first exon of zebrafish mbp (-1794 to +159 bp from the transcription start site) was synthesized by Invitrogen (Carlsbad, CA, USA). The coding region of mCitrine was amplified by polymerase chain reaction using pCS2 + 8NmCitrine (Addgene, Cambridge, MA, USA) as the template. These DNA fragments were cloned into Tol2 vector (Kawakami, 2007) using the In-fusion HD cloning kit (Takara Bio, Shiga, Japan) to make a circular plasmid (pT2-mbp-mCitrine). The pT2-mbp-mCitrine plasmid and transposase mRNA (Kawakami, 2007) were injected into zebrafish embryos at the 1- to 4-cell stage. Larval zebrafish expressing mCitrine in the spinal cord were selected and maintained. Mature F0 zebrafish were mated with albino zebrafish, and F1 zebrafish expressing mCitrine in the spinal cord were selected and maintained. Mature F1 zebrafish were mated with Tg (eno2: Cerulean) zebrafish, which express Cerulean fluorescent protein specifically in neurons (Bai et al., 2007; Sasagawa et al., 2016a), to create Tg (mbp: mCitrine, eno2: Cerulean) zebrafish. Double Tg zebrafish expressing mCitrine and Cerulean in the spinal cord were selected and subsequently maintained and bred according to previously described methods (Westerfield, 2007; Nishimura et al., 2016). Briefly, zebrafish were raised at 28.5 ± 0.5°C with a 14-h/10-h light/dark cycle. Embryos were obtained via natural mating and cultured in fish medium (0.07 mM KCl, 2 mM CaCl_2_, 0.5 mM MgSO_4_, and 0.7 mM NaHCO_3_, pH 7.4) until 5 days post-fertilization (dpf), at which point they were used for *in vivo* imaging analysis. Zebrafish were maintained on living *Paramecium* spp. from 5 dpf.

### *In Vivo* Imaging of Tg (mbp: mCitrine, eno2: Cerulean) Zebrafish

Tg (mbp: mCitrine, eno2: Cerulean) zebrafish were exposed to chemicals from 10 h post-fertilization to 5 dpf in 12-well plates (20 larvae/well). At 5 dpf, zebrafish were anesthetized with 2-phenoxyethanol and placed in a 96-well imaging plate (ZF plate, Hashimoto Electric Industry, Mie, Japan). *In vivo* imaging and quantitative analysis of the mCitrine fluorescent signal was performed using ImageXpress Micro with customized programs (Molecular Device, Sunnyvale, CA, USA). Briefly, the image was first processed to identify the region of hindbrain and spinal cord using the eno2 promoter-driven Cerulean fluorescence signal (green borders shown in **Figures 4** and **5**). The mbp promoter-driven mCitrine fluorescence signals within this region were then measured (red dots shown in **Figures 4** and **5**). Bright-field images of zebrafish were captured using an SMZ25 microscope (Nikon, Tokyo, Japan).

### Quantitative PCR Analysis

Total RNA was extracted from zebrafish at 5 dpf using a Nucleospin RNA XS kit (Takara, Kyoto, Japan) according to the manufacturer’s protocol. cDNAs were generated using a ReverTra Ace qPCR RT Kit (Toyobo). qPCR was performed using an ABI Prism 7300 PCR system (Life Technologies, Carlsbad, CA, USA) with THUNDERBIRD SYBR qPCR Mix (Toyobo). The thermal cycling conditions were: 95°C for 1 min, followed by 40 cycles of 95°C for 15 s, 60°C for 15 s, and 72°C for 45 s. We measured the expression of 3-hydroxy-3-methylglutaryl-CoA reductase (*hmgcr*), 7-dehydrocholesterol reductase (*dhcr7*), mbp, and eukaryotic translation elongation factor 1 alpha 1 (*ef1a*). *hmgcr*, *dhcr7*, *and mbp* mRNA levels were normalized to *ef1a* mRNA levels to correct for variability in the initial template concentration and the conversion efficiency of the reverse transcription reaction. The primer sequences are shown in Supplementary Table S6.

### Statistical Analysis

Statistical analysis was performed using Prism 6 (GraphPad, La Jolla, CA, USA). Group means were compared by analysis of variance. Alpha was set at 0.05. Dunnett’s and Tukey’s multiple comparisons tests were used for *post hoc* analysis of the *in vivo* imaging and qPCR data, respectively, when significant effects were found by analysis of variance. Data are shown as the mean ± standard error (SEM).

## Results

### Identification of Differentially Expressed Genes Regulated by Both Miconazole and Clobetasol

**FIGURE 1:**
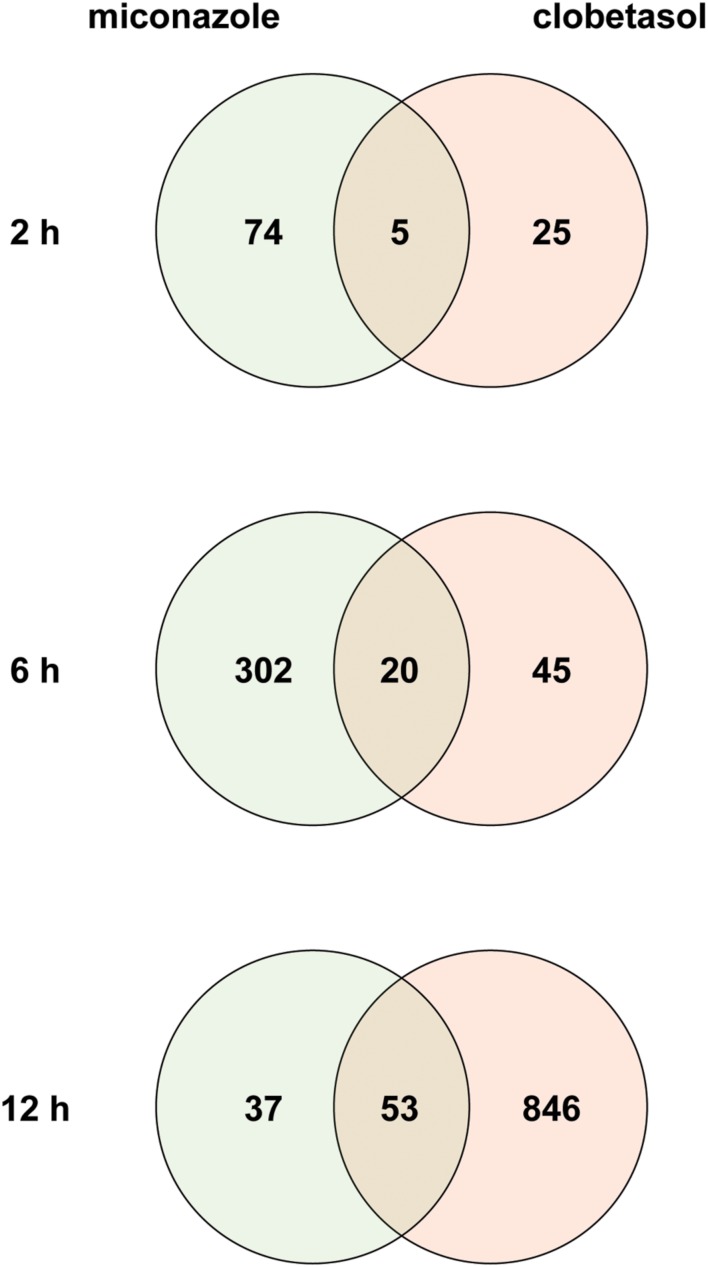
**Venn diagrams of differentially expressed genes in mEpiSC-OPCs treated with miconazole or clobetasol compared with control mEpiSC-OPCs.** Transcriptome data from mEpiSC-OPCs treated with miconazole or clobetasol (GSE63804) were downloaded from a public database. Genes differentially expressed in control mEpiSC-OPCs versus mEpiSC-OPCs treated with miconazole or clobetasol for 2, 6, or 12 h were identified using a false discovery rate of 20% as the threshold. The number of DEGs in each group and the overlap between groups are shown in the Venn diagrams for 2, 6, and 12 h of treatment.

To identify common mechanisms underlying myelination induced by miconazole and clobetasol, we downloaded a transcriptome dataset from an analysis of the effects of the two drugs on mEpiSC-OPCs (Najm et al., 2015) from a public database (Barrett et al., 2009). Using a false discovery rate of 20% as the threshold, we identified 79 and 30 DEGs in mEpiSC-OPCs treated for 2 h with miconazole and clobetasol, respectively [**Figure 1**, Supplementary Tables S1(1,2)]. Five DEGs were common to both treatments (Supplementary Table S2). We also identified 322 and 65 DEGs in mEpiSC-OPCs treated for 6 h with miconazole and clobetasol, respectively [**Figure 1**, Supplementary Tables S1(3,4)]. Twenty DEGs were common to both treatments (Supplementary Table S2). We also identified 90 and 899 DEGs in mEpiSC-OPCs treated for 12 h with miconazole and clobetasol, respectively [**Figure 1**, Supplementary Tables S1(5,6)]. Fifty-three DEGs were common to both treatments (Supplementary Table S2). The change in expression of these common DEGs was the same, with the exception of *Abca1* that was downregulated and upregulate by miconazole and clobetasol, respectively (Supplementary Table S2). Taken together, these data suggest that genes dysregulated in both miconazole- and clobetasol-treated cells may be involved in a common mechanism to promote myelination.

### Identification of Cholesterol Biosynthesis as the Key Biological Pathway Enriched in Genes Regulated by Both Miconazole and Clobetasol

**FIGURE 2:**
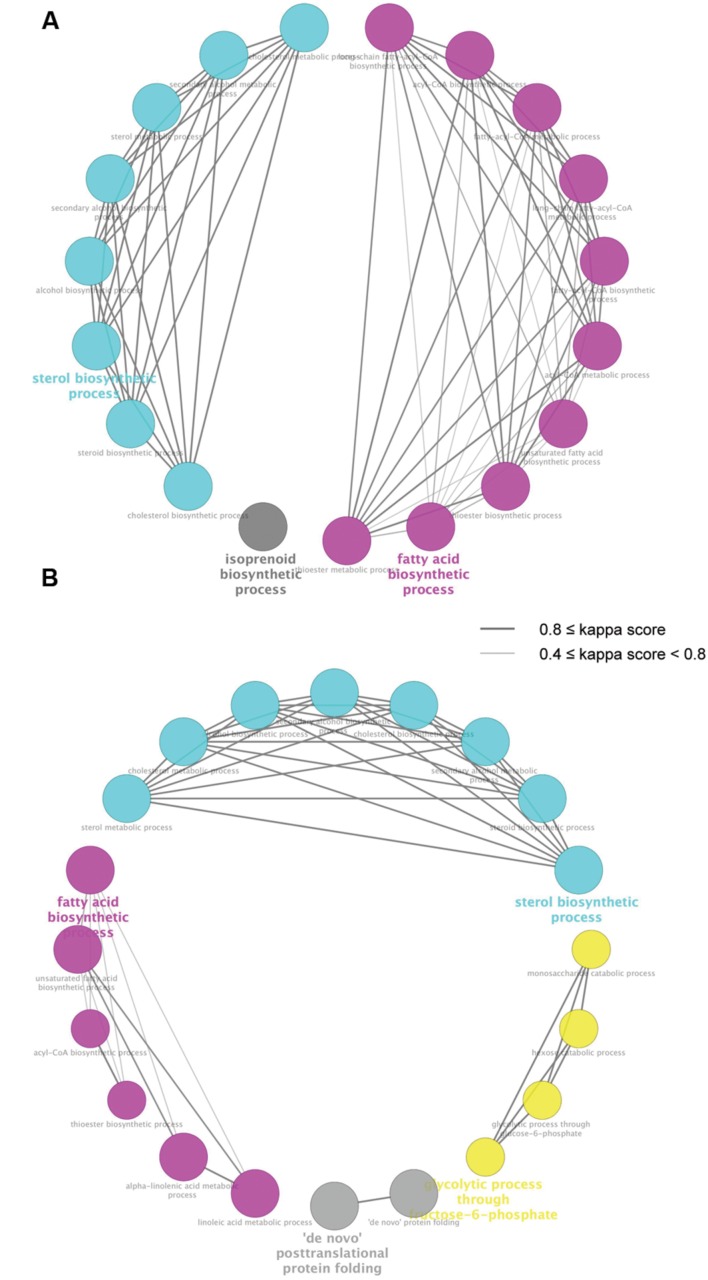
**Biological pathways significantly enriched in genes regulated by both miconazole and clobetasol.** DEGs common to mEpiSC-OPCs treated with miconazole or clobetasol for 6 or 12 h were independently subjected to ClueGO using Gene Ontology Biological Pathway as the database. The pathways significantly enriched in the common DEGs at 6 and 12 h treatment are shown in **(A,B)**, respectively. Each circle represents one biological pathway. Pairs of biological pathways with similar kappa scores are connected by lines. Biological pathways clustered in the same group are shown in the same color.

To identify biological processes enriched in the DEGs regulated by both miconazole and clobetasol, we used ClueGO, a bioinformatics tool that has been used successfully to identify biological functions associated with given gene sets (Bindea et al., 2009; Sasagawa et al., 2016b). ClueGO identified 19 and 20 biological pathways significantly enriched in the 20 (6 h) and 53 (12 h) DEGs, respectively, regulated by both miconazole and clobetasol [**Figure 2**, Supplementary Tables S3(1,2)]. There were no biological pathways significantly enriched in the five DEGs regulated in common by both miconazole and clobetasol at 2 h, possibly because of the small number of DEGs. The 19 biological pathways identified at 6 h were clustered into three groups; sterol biosynthetic process, fatty acid biosynthetic process, and isoprenoid biosynthetic process [Supplementary Table S3(1)]. The 20 biological pathways at 12 h were clustered into four groups; sterol biosynthetic process, fatty acid biosynthetic process, “*de novo*” posttranslational protein folding, and glycolytic process through fructose-6-phosphate [Supplementary Table S3(2)]. These results suggest that sterol biosynthesis and fatty acid biosynthetic processes may be key pathways involved in miconazole- and clobetasol-stimulated myelination.

### Identification of SREBFs as Important Transcription Factors Regulating the Expression of Differentially Expressed Genes Common to Miconazole and Clobetasol

**FIGURE 3:**
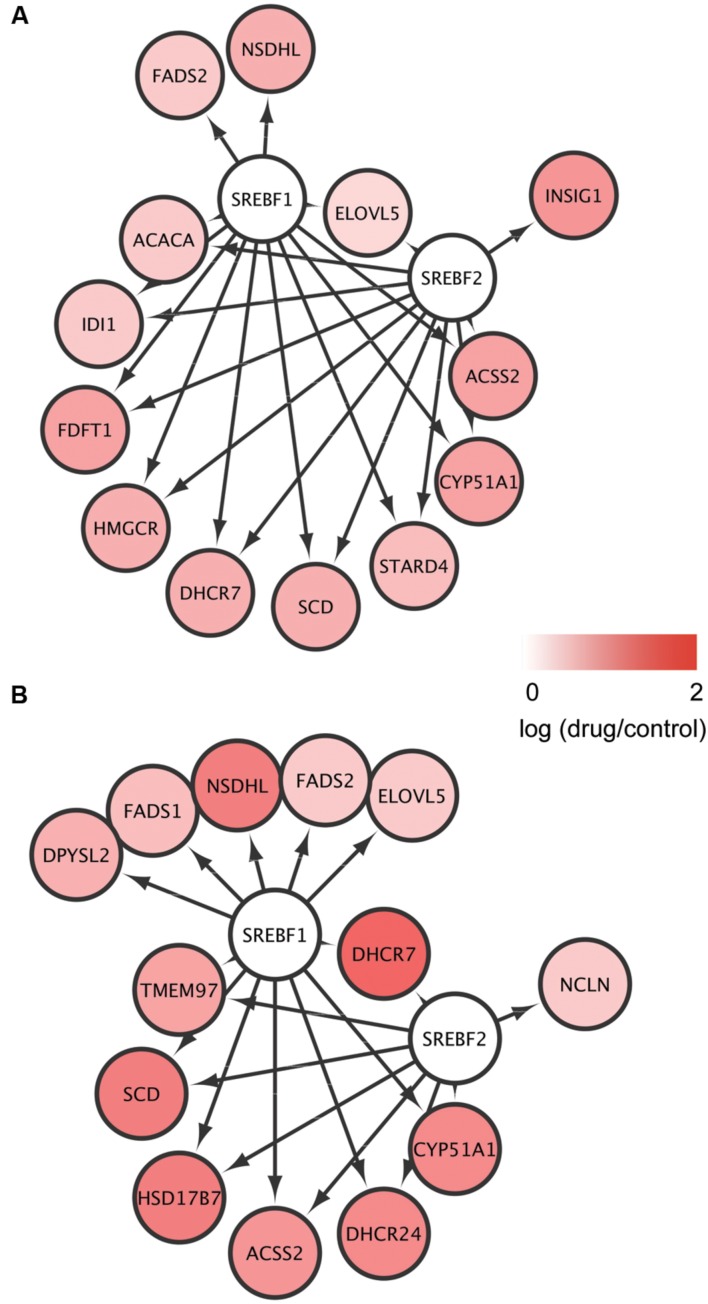
**Identification of SREBFs as key transcription factors for the genes regulated by both miconazole and clobetasol.** **(A,B)** DEGs common to mEpiSC-OPCs treated with miconazole or clobetasol for 6 or 12 h were independently subjected to iRegulon to identify TFs potentially regulating the DEGs. The DEGs potentially regulated by SREBF1 and SREBF2 after 6 h **(A)** and 12 h **(B)** treatment are shown.

To identify TFs potentially regulating the DEGs common to mEpiSC-OPCs treated with miconazole or clobetasol, we used iRegulon, which has been used successfully to identify important TFs from given gene sets (Nishimura et al., 2015b; Sasagawa et al., 2016a,b). Based on the list of common DEGs at 6 and 12 h (Supplementary Table S2), the iRegulon analysis identified SREBF1 and SREBF2 [**Figure 3**, Supplementary Tables S4(1,2)], which is consistent with previous studies showing that SREBF activation increases the expression of 3-hydroxy-3-methylglutaryl-CoA reductase (*HMGCR*) (Vallett et al., 1996; Bennett et al., 2004), stearoyl-CoA desaturase (*SCD*) (Tabor et al., 1999), cytochrome P450 family 51 subfamily A polypeptide 1 (*CYP51A1*) (Halder et al., 2002), acyl-CoA synthetase short-chain family member 2 (*ACSS2*) (Ikeda et al., 2001), and 7-dehydrocholesterol reductase (*DHCR7*) (Prabhu et al., 2014). SREBFs are also known to be important regulators of sterol biosynthesis (Ye and DeBose-Boyd, 2011) and myelination (Norrmen et al., 2014). There were no TFs significantly enriched in the five DEGs regulated by both miconazole and clobetasol for 2 h, probably owing to the limited number of DEGs. These results suggest that activation of SREBFs and the subsequent increase in expression of these SREBF target genes may be involved in the pro-myelinating effects of miconazole and clobetasol.

### Identification of Fenofibrate as a Drug That Increases the Expression of Genes Regulated by SREBFs

From our findings with miconazole and clobetasol, we hypothesized that chemicals that increase the expression of the SREBF target genes might stimulate myelination. To test this, we searched the Comparative Toxicogenomics Database (Davis et al., 2015) for chemicals able to upregulate expression of *SCD*, identified as the common DEG at 2, 6, and 12 h, and of *HMGCR*, identified as the common DEG at 2 and 6 h (**Figure 2**, Supplementary Table S2). The Comparative Toxicogenomics Database is a public database of the relationships between chemicals and various parameters, including gene expression, curated from the scientific literature. This *in silico* screening identified nine FDA-approved drugs that increase expression of both *SCD* and *HMGCR* [Supplementary Table S5(3)]. It is noteworthy that the list includes clozapine and haloperidol, two antipsychotic medications used to treat schizophrenia. Both compounds are known to activate SREBF and upregulate the transcription of SREBF target genes (Ferno et al., 2006). The PPARα agonist fenofibrate has also been shown to activate SREBF2 (Rampler et al., 2003) and to have therapeutic potential for the treatment of adrenoleukodystrophy (Berger et al., 2010), consistent with the possibility that this drug may stimulate myelination.

### Fenofibrate and Gemfibrozil Increase Expression of a Fluorescent Reporter Protein Regulated by the Myelin Basic Protein Promoter in Zebrafish

**FIGURE 4:**
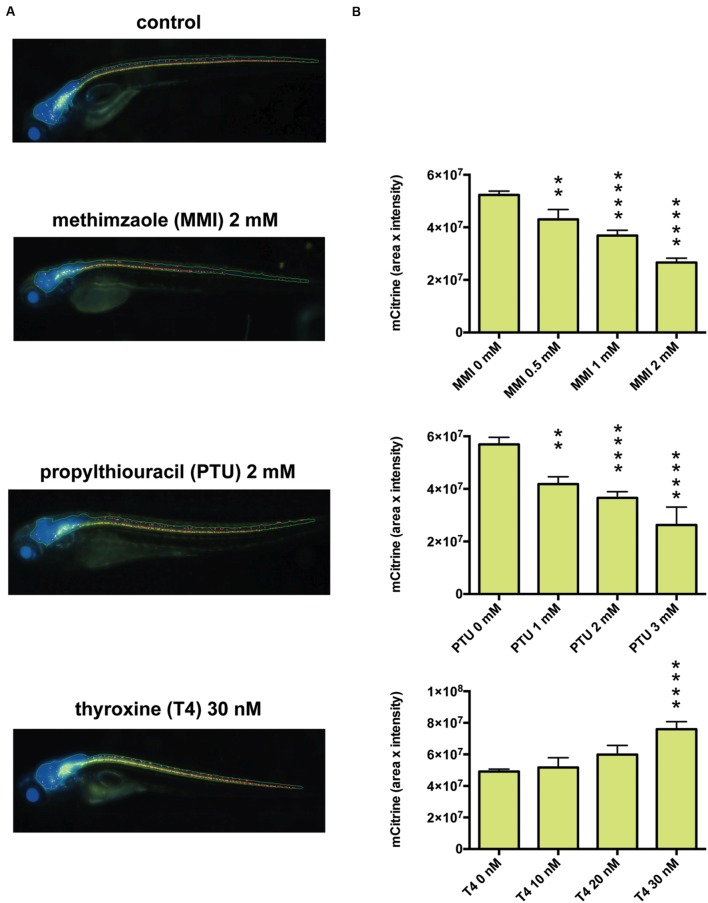
**Effects of thyroid hormone modulators on mbp promoter-driven fluorescence in zebrafish.** **(A)** Representative images from *in vivo* analysis of Tg (mbp: mCitrine, eno2: Cerulean) zebrafish incubated with or without the indicated concentrations of methimazole (MMI), propylthiouracil (PTU), or thyroxine (T4). **(B)** Quantification of mCitrine fluorescence intensity within the area of Cerulean fluorescence. (Top) Zebrafish were untreated (*n* = 40) or treated with MMI (*n* = 16 for 0.5 mM, *n* = 26 for 1 mM, *n* = 27 for 2 mM). (Middle) Zebrafish were untreated (*n* = 11) or treated with PTU (*n* = 5 for 1 mM, *n* = 10 for 2 mM, *n* = 3 for 3 mM). (Bottom) Zebrafish were untreated (*n* = 20) or treated with T4 (*n* = 6 for 10 nM, *n* = 5 for 20 nM, *n* = 9 for 30 nM). ^∗∗^*p* < 0.01, ^∗∗∗∗^*p* < 0.0001 compared with control.

To investigate whether fenofibrate and a second PPARα agonist, gemfibrozil, could increase myelin expression *in vivo*, we examined Tg (mbp: mCitrine, eno2: Cerulean) zebrafish, which express two fluorescent reporters: Cerulean in neurons (driven by the eno2 promoter) and mCitrine in oligodendrocytes (driven by the mbp promoter). MBP is the major constituent of the myelin sheath and is produced by oligodendrocytes. To validate *in vivo* fluorescent imaging of zebrafish as an assay for examining the effects of chemicals on CNS myelination, we first treated the animals with methimazole (MMI) and propylthiouracil (PTU), which are known to decrease CNS myelination, or with thyroxine, which increases it (Jagannathan et al., 1998; Gilbert et al., 2012). To quantify the effects on myelin promoter activity, we measured the fluorescence intensity of mCitrine within the Cerulean-positive area of the CNS in living zebrafish and found that MMI and PTU dose-dependently decreased the mCitrine signal, whereas thyroxine dose-dependently increased it (**Figure 4**). These results suggest that *in vivo* imaging of mbp promoter-driven mCitrine expression in zebrafish can be used to assess the effects of chemicals on myelination.

**FIGURE 5:**
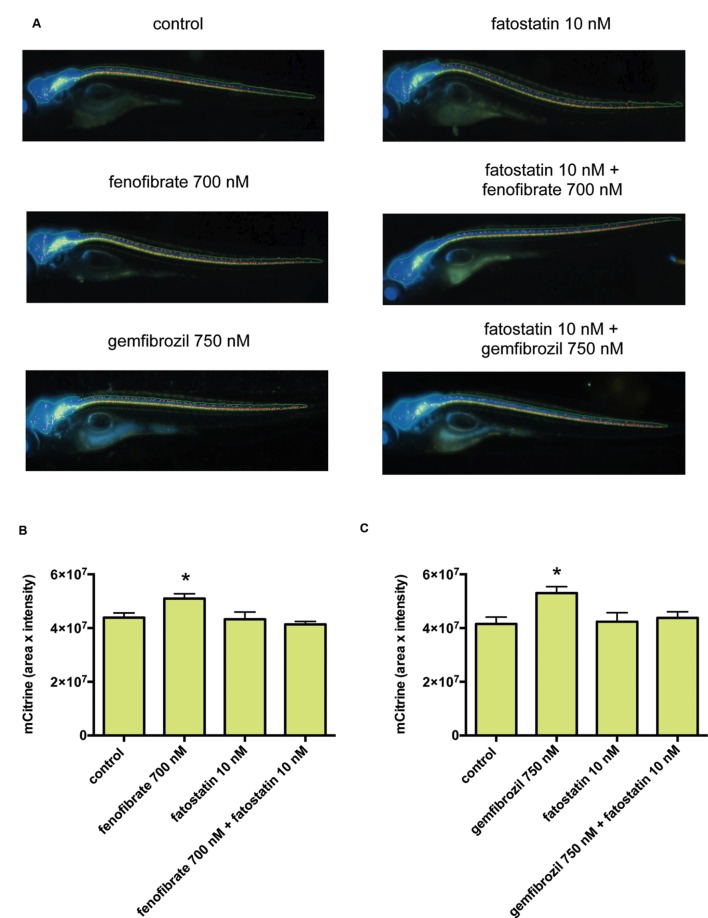
**Fenofibrate and gemfibrozil increase mbp promoter-driven fluorescent reporter expression through activation of SREBFs.** **(A)** Representative images from *in vivo* analysis of Tg (mbp: mCitrine, eno2: Cerulean) zebrafish incubated with or without the indicated concentrations of fenofibrate, gemfibrozil, and fatostatin. **(B,C)** Quantification of mCitrine fluorescence intensity within the area of Cerulean fluorescence. **(B)** Zebrafish were untreated (*n* = 28) or treated with fenofibrate (*n* = 27), fatostatin (*n* = 20), or fenofibrate and fatostatin (*n* = 5). ^∗^*p* < 0.05 compared with control. **(C)** Zebrafish were untreated (*n* = 7) or treated with gemfibrozil (*n* = 7), fatostatin (*n* = 8), or gemfibrozil and fatostatin (*n* = 8). ^∗^*p* < 0.05 compared with control.

We then examined the effects of fenofibrate and gemfibrozil on the transgenic zebrafish and found that both fenofibrate and gemfibrozil significantly increased the mCitrine fluorescence (**Supplementary Figure S1**). To determine the involvement of SREBFs in this increase, we co-treated zebrafish with fenofibrate or gemfibrozil and fatostatin, a specific inhibitor of SREBFs (Kamisuki et al., 2009). Notably, fatostatin reduced the mCitrine fluorescence signal in zebrafish treated with fenofibrate or gemfibrozil to the level seen in control zebrafish (**Figure 5**). To exclude the possibility that the changes in mCitrine fluorescence result from drug toxicity, we examined the zebrafish by bright-field microscopy. As shown in **Supplementary Figure S2**, the drug-treated animals showed no malformations, except in the presence of MMI and PTU at 2 mM. Thus, it is unlikely that the observed changes in mCitrine fluorescence are due to drug-related toxicity. To examine whether SREBFs may be activated in zebrafish treated with fenofibrate or gemfibrozil, we quantified expression of the SREBF target genes *hmgcr* and *dhcr7*. As shown in **Supplementary Figure S3**, *hmgcr* mRNA levels were significantly increased in zebrafish treated with either fenofibrate or gemfibrozil. *dhcr7* mRNA levels were also increased by both drugs, but the change was significant only in gemfibrozil-treated zebrafish. These results suggest that both fenofibrate and gemfibrozil may activate SREBFs in zebrafish. We also quantified the expression of mbp mRNA to determine its association with the increase in mCitrine fluorescence in fenofibrate- and gemfibrozil-treated zebrafish. As shown in **Supplementary Figure S3**, mbp mRNA levels were increased by both drugs, with gemfibrozil having a statistically significant effect. These results suggest that the fenofibrate- and gemfibrozil-induced increases in mCitrine fluorescence may correlate positively with the increase in mbp mRNA. Taken together, these results suggest that fenofibrate and gemfibrozil may stimulate myelination through activation of SREBFs in zebrafish.

## Discussion

In this study, we demonstrated that activation of SREBFs might be involved in myelination induced by miconazole and clobetasol. We also demonstrated that fenofibrate and gemfibrozil increase mbp promoter-driven expression of a fluorescent reporter protein in zebrafish in a SREBF-dependent manner, suggesting that the pro-myelinating effects of fenofibrate and gemfibrozil occur through SREBF activation.

### Myelination Is Induced by Activation of SREBFs

Using comparative transcriptome analysis, we demonstrated that SREBF activation may be a common mechanism underlying myelination stimulated by miconazole and clobetasol. These results are consistent with previous reports supporting a role for SREBFs in myelination. For example, mammalian target of rapamycin complex 1 (mTORC1) regulates oligodendrocyte differentiation and myelination through activation of SREBFs (Norrmen et al., 2014), and mice carrying mutations in SREBF cleavage activating protein (SCAP), a SREBF activator, show congenital hypomyelination (Verheijen et al., 2009). Although SREBFs have not previously been associated with the pharmacological effects of miconazole and clobetasol, the transcriptome analysis revealed cholesterol biosynthesis as a pathway significantly enriched in genes perturbed by both drugs (Najm et al., 2015). Collectively, our data and these observations strongly suggest that the pro-myelinating effects of miconazole and clobetasol are dependent on SREBF activation.

We also demonstrated that known SREBF target genes, including *HMGCR* (Vallett et al., 1996; Bennett et al., 2004), *SCD* (Tabor et al., 1999), *CYP51A1* (Halder et al., 2002), *ACSS2* (Ikeda et al., 2001), and *DHCR7* (Prabhu et al., 2014), are differentially upregulated in mEpiSC-OPCs treated with both miconazole and clobetasol. Expression of SCD positively correlates with peripheral axonal myelination (Garbay et al., 1998). Increased HMGCR expression is the predominant mechanism by which myelination is induced by neuregulin 1 in Schwann cells (Pertusa et al., 2007). These findings suggest that increased expression of *SCD* and *HMGCR* may stimulate myelination.

Using *in silico* screening of the Comparative Toxicogenomics Database (Davis et al., 2015), we identified nine FDA-approved drugs that increase the expression of both *SCD* and *HMGCR*.

Clozapine and haloperidol are known to increase *SCD* and *HMGCR* expression through activation of SREBFs (Ferno et al., 2006), and clozapine also promotes myelin lipid synthesis in cultured oligodendrocytes (Steiner et al., 2014). Progesterone stimulates remyelination in mouse models of demyelinating diseases (Chew and DeBoy, 2015). Amiodarone increases phospholipid levels, possibly through induction of genes associated with cholesterol synthesis (Antherieu et al., 2011). Although we are unaware of previous reports of a relationship between the PPARγ agonist troglitazone and myelination, one study showed that another PPARγ agonist, pioglitazone, does stimulate myelination (Eto et al., 2008). Thus, chemicals that can activate SREBFs and increase the expression of *SCD* and *HMGCR* may stimulate myelination.

### Fenofibrate and Gemfibrozil Stimulate Myelination by Activating SREBFs

We used zebrafish to examine the effects of fenofibrate and gemfibrozil on myelination *in vivo*. In a survey of 1318 human drug targets, 86% had orthologs in zebrafish (Gunnarsson et al., 2008). This high conservation makes the zebrafish a useful animal model for drug screening. Although there can be interspecies differences in drug pharmacodynamics, several studies have shown that zebrafish can be used successfully to identify novel drugs for treatment of human diseases and to examine the safety and toxicity of drugs in preclinical studies (Barros et al., 2008; Helenius and Yeh, 2012; Mathias et al., 2012; Rihel and Schier, 2012; Esterberg et al., 2013). Zebrafish have also been used as a model organism to investigate CNS myelination (Mathews et al., 2014; Preston and Macklin, 2015). The structure of myelin and the molecular mechanism underlying myelination are well conserved between zebrafish and mammals (Preston and Macklin, 2015). The ease with which zebrafish can be genetically manipulated, their transparency, and their ability to absorb a wide range of chemicals from the surrounding medium make zebrafish a highly suitable model for *in vivo* chemical screening (Nishimura et al., 2015a; Rennekamp and Peterson, 2015). Indeed, zebrafish have been used successfully to identify novel compounds with pro-myelinating properties (Buckley et al., 2010). Using *in vivo* imaging of zebrafish, we were able to demonstrate that fenofibrate and gemfibrozil increased the activity of the mbp promoter via activation of SREBFs.

Several mechanisms can be proposed by which fenofibrate and gemfibrozil might activate SREBFs. Both compounds are PPARα agonists (Friedland et al., 2012), and activation of PPARα can stimulate SREBF signaling through multiple mechanisms, including increasing SREBF expression (Rampler et al., 2003; Martens et al., 2008; Yan et al., 2014), enhancing SREBF proteolytic cleavage (Knight et al., 2005), and increasing SREBF activity via recruitment of transcriptional co-activators (van der Meer et al., 2010). Gemfibrozil activates PPARβ/δ (Ogata et al., 2009; Roy et al., 2013) and increases the expression of myelin in human oligodendrocytes through PPARβ/δ activation (Jana et al., 2012). PPARβ/δ activation can also stimulate SREBF signaling (Yang et al., 2014). Fibrates have been considered as potential therapeutics for diseases associated with impaired oligodendrocytes, such as multiple sclerosis, adrenoleukodystrophy, schizophrenia, and traumatic brain injury (Besson et al., 2005; Yang et al., 2008; Berger et al., 2010; Rolland et al., 2012; Mandrekar-Colucci et al., 2013). Further studies are required to determine whether fenofibrate and gemfibrozil can stimulate myelination through activation of SREBFs in these neurological disorders.

## Author Contributions

YN conceived the study, performed the bioinformatics analysis, generated the transgenic zebrafish, and wrote the paper. YA performed experiments to validate the effects of chemicals on myelination in zebrafish. SO analyzed the transcriptome data. RK generated the transgenic zebrafish. SS, SM, MY, SO, and KK provided assistance with experiments. TT conceived the study and wrote the paper.

## Conflict of Interest Statement

The authors declare that the research was conducted in the absence of any commercial or financial relationships that could be construed as a potential conflict of interest.

The reviewer MB and handling Editor declared their shared affiliation, and the handling Editor states that the process nevertheless met the standards of a fair and objective review.

## Supplementary Material

**FIGURE S1 | Effects of fenofibrate and gemfibrozil on mbp promoter-driven fluorescence in zebrafish.** **(A,B)** Quantification of mCitrine fluorescence intensity within the area of Cerulean fluorescence. **(A)** Zebrafish were untreated (*n* = 9) or treated with fenofibrate (*n* = 12 for 100 nM, *n* = 7 for 400 nM, *n* = 15 for 700 nM). **(B)** Zebrafish were untreated (*n* = 17) or treated with gemfibrozil (*n* = 11 for 250 nM, *n* = 6 for 500 nM, *n* = 14 for 750 nM). ^∗∗^*p* < 0.01, ^∗∗∗^*p* < 0.001 compared with control.

**FIGURE S2 | Bright-field images of zebrafish treated with various drugs.** Zebrafish were treated with the indicated drugs from 10 h post-fertilization to 5 dpf and bright-field images were captured at 5 dpf.

**FIGURE S3 | Quantitative PCR (qPCR) analysis of *hmgcr*, *dhcr7*, and *mbp* in zebrafish treated with fenofibrate or gemfibrozil.** qPCR analysis of *hmgcr*, *dhcr7*, and *mbp* mRNA levels in zebrafish treated with or without fenofibrate or gemfibrozil from 10 h post-fertilization to 5 dpf. Expression was normalized to *ef1a* mRNA levels. *n* = 6 per group. If two groups do not share a letter, the difference between them is significant.

## Abbreviations

DEG: differentially expressed gene
dpf: days-post-fertilization
mbp: myelin basic protein
mEpiSC: murine epiblast stem cell
OPC: oligodendrocyte progenitor cell
PPAR: peroxisome proliferator-activated receptor
SREBF: sterol regulatory element binding factor
TF: transcription factor
