# Supplementary figures and images for: Activation of Sterol Regulatory Element Binding Factors by Fenofibrate and Gemfibrozil Stimulates Myelination in Zebrafish
Source: Front Pharmacol. 2016 Jul 11;7:206. doi: 10.3389/fphar.2016.00206 (PMC4939524; doi:10.3389/fphar.2016.00206)

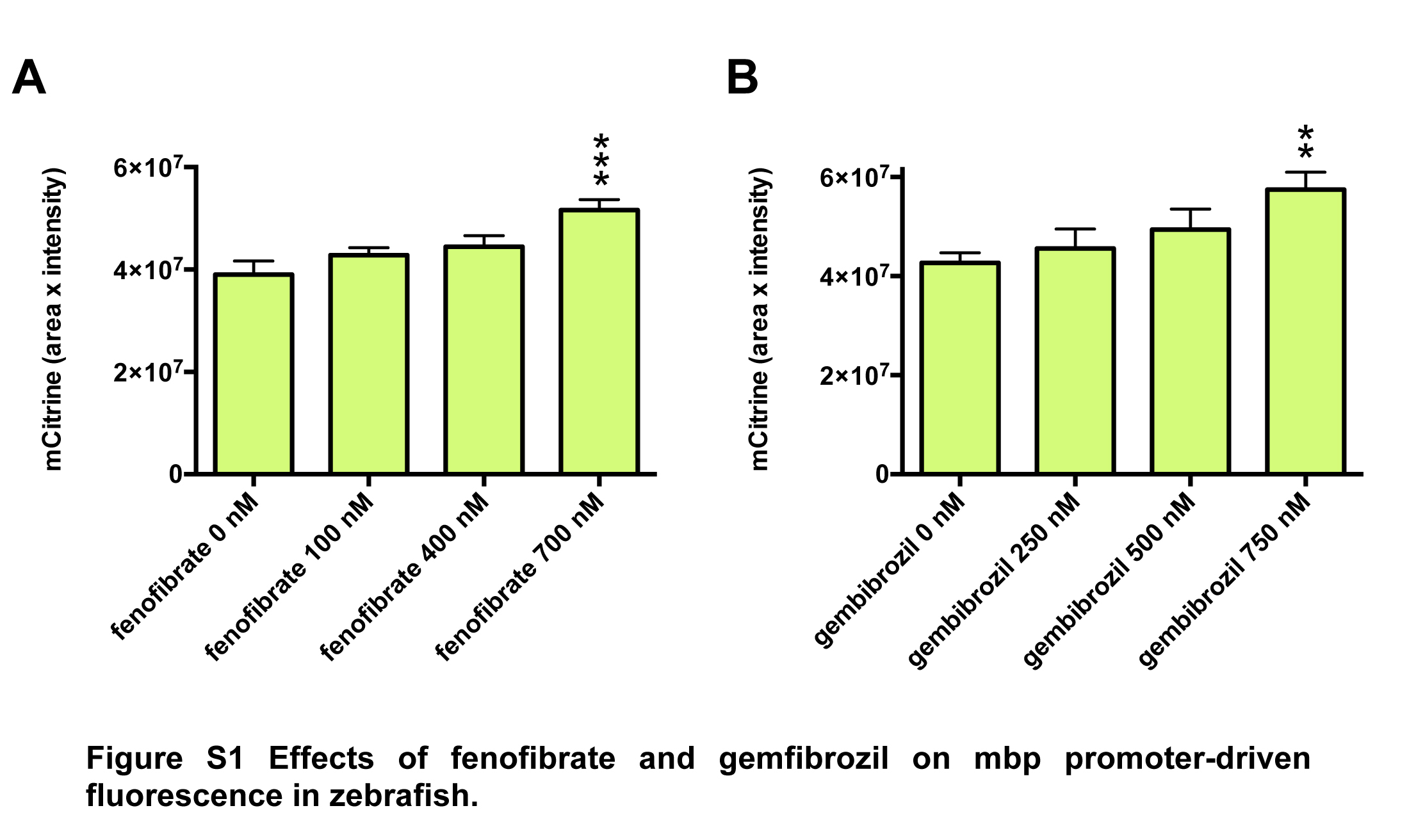

Supplement: Supplementary file 1 [file Image_1.JPG]

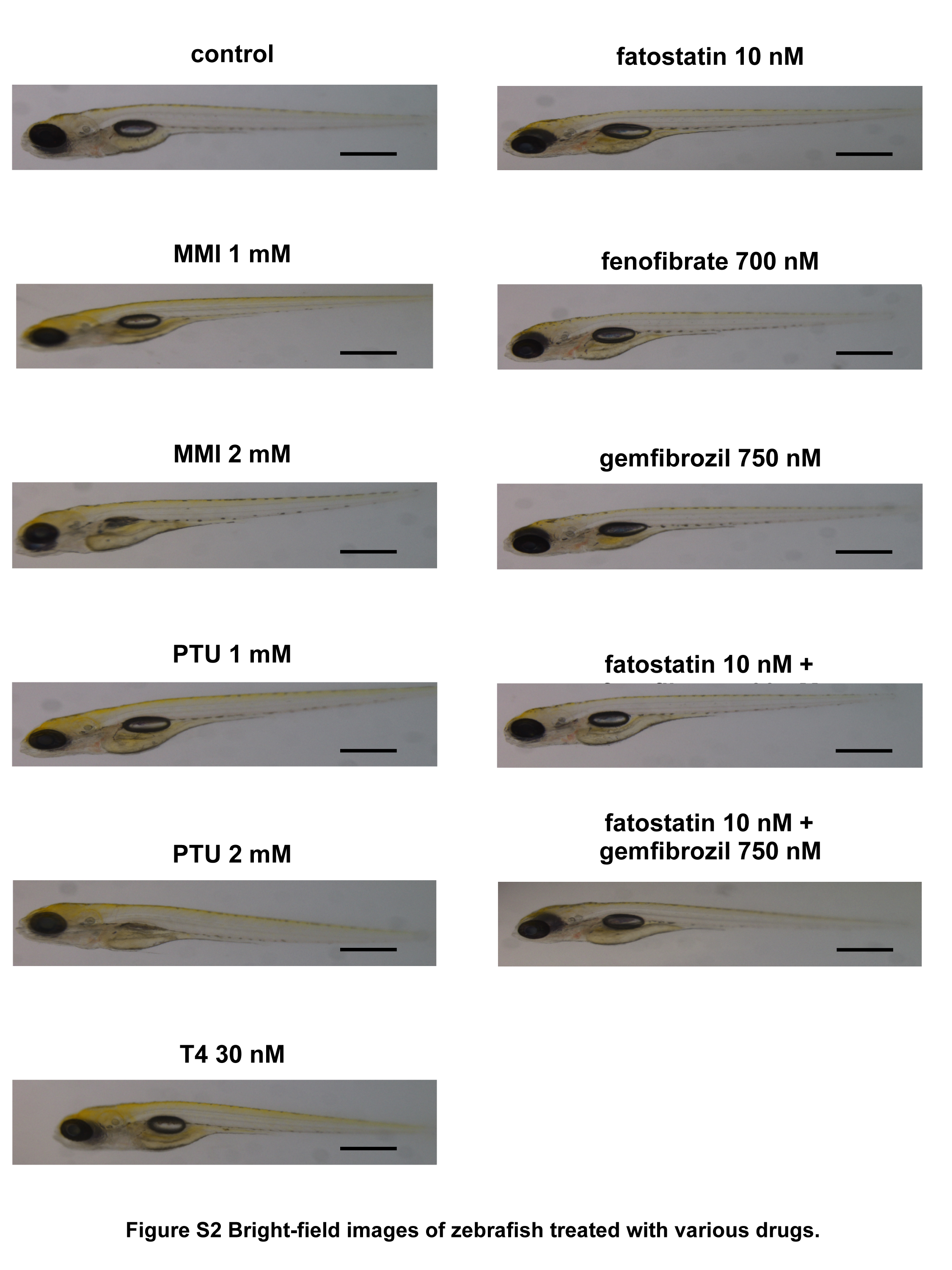

Supplement: Supplementary file 2 [file Image_2.JPG]

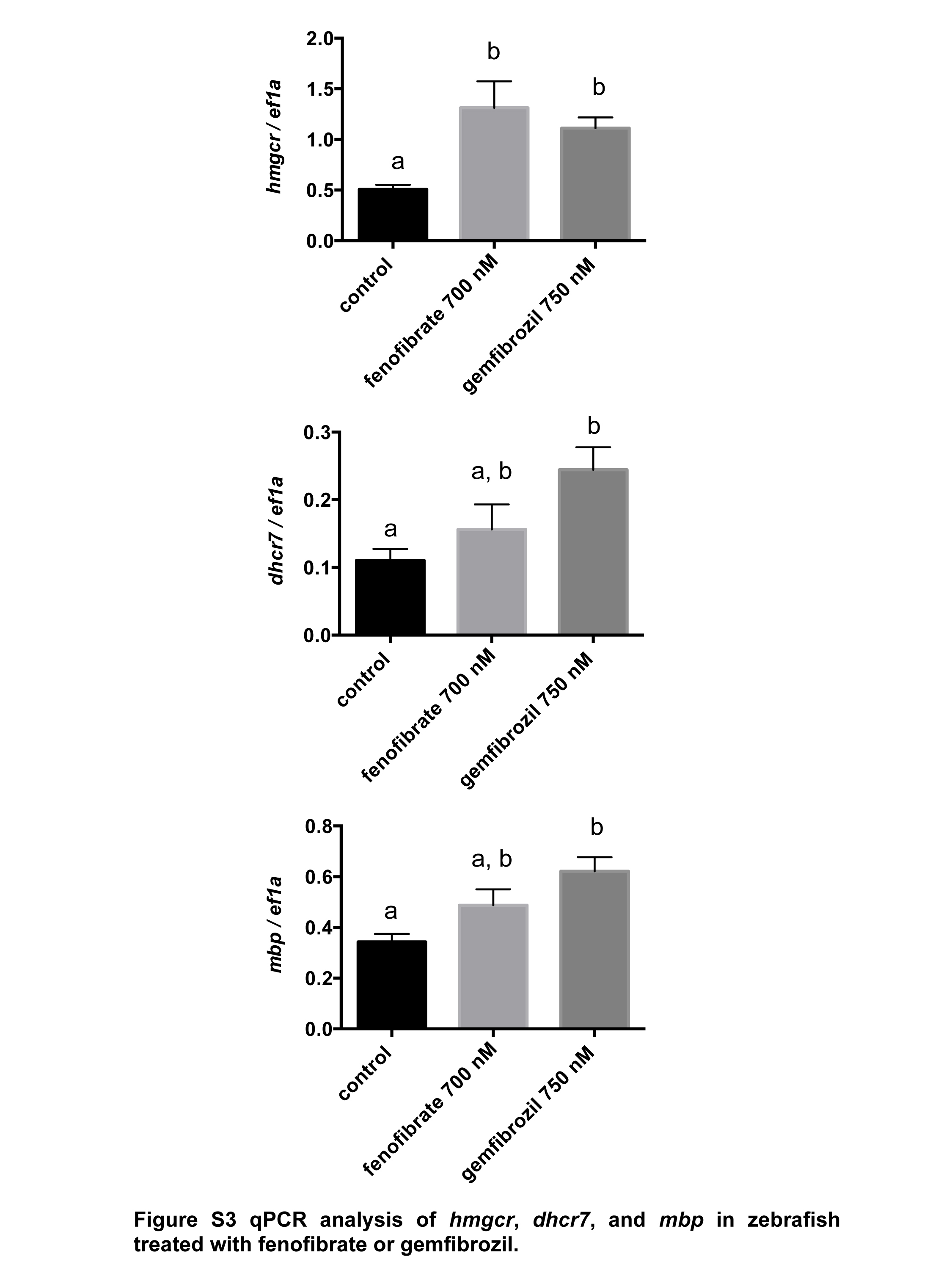

Supplement: Supplementary file 3 [file Image_3.JPG]
